# Fabrication of Core–Shell Aggregates from Reclaimed Asphalt Pavement (RAP): A Modification Strategy for Tailoring Structural and Surface Properties

**DOI:** 10.3390/ma18245542

**Published:** 2025-12-10

**Authors:** Qingsong Chen, Qinhao Deng, Shaopeng Wu, An Liu, Guoxin Xia

**Affiliations:** State Key Laboratory of Silicate Materials for Architectures, Wuhan University of Technology, Wuhan 430070, China

**Keywords:** reclaimed asphalt pavement (RAP), core–shell structure, cement hydration, particle size regulation, response surface methodology, surface modification

## Abstract

**Highlights:**

**What are the main findings?**

- Optimized synthesis reduced aggregate crushing value by 43.9%.
- Core–shell RAP aggregates were fabricated via cement hydration.

**What are the implications of the main findings?**

- The method resolves issues such as RAP “pseudo-gradation” and excess fine content.
- It enables high-content RAP use in high-grade pavement layers.

**Abstract:**

This study presents a modification strategy to fabricate core–shell composite aggregates from reclaimed asphalt pavement (RAP), aligning with green chemistry principles for waste valorization. The method involves creating a porous cementitious shell on the surface of RAP particles through a controlled hydration process. This surface modification simultaneously addresses the inherent structural weaknesses and irregular morphology of raw RAP, enabling the design of materials with desired properties. A face-centered central composite design (FCCD) was employed to optimize the synthesis process, elucidating the nonlinear relationships between key synthesis parameters and the final material characteristics. The optimized synthesis yielded porous aggregates with significantly enhanced structural integrity, evidenced by a 43.9% reduction in crushing value. Furthermore, the surface modification effectively regulated the material’s morphology and particle size distribution, leading to a 3.6 mm increase in median particle size (D_50_) and a 27.69% decrease in the content of fines (<4.75 mm). Microstructural characterization confirmed the formation of a rough, porous cementitious shell composed of hydration products, which provides the structural basis for the material’s enhanced performance. This work establishes a clear structure–property relationship, demonstrating a new pathway for the rational design and synthesis of functional porous materials from solid waste for application in high-grade pavements.

## 1. Introduction

The heavy reliance on non-renewable virgin aggregates and the continuous accumulation of reclaimed asphalt pavement (RAP) pose significant challenges to the sustainable development of highway engineering. The combined pressures of resource depletion, soaring extraction costs, and tightening environmental policies compel the industry to seek greener solutions for road construction [1,2,3].

Although RAP recycling is essential for sustainability, high-content application faces two primary technical bottlenecks: material aging and gradation distortion [4,5]. Firstly, the aged binder and weakened aggregates fail to meet the mechanical demands of new pavements [6,7,8]. Secondly, milling generates excessive fines and “pseudo-particles”, causing compositional variability that intensifies with higher RAP content [9,10]. Additionally, the temperature differential between preheated RAP (limited to prevent secondary aging) and superheated virgin aggregates impedes effective binder blending, further compromising mixture performance [11,12], such as strength and moisture resistance [13].

In response to these issues, various strategies have been explored. To counteract performance degradation, methods include reducing fine content [14], incorporating polymers [15] and fibers [16], using rejuvenators [17,18], or composite modification [19]. Gradation distortion is addressed through fractionation [20,21,22,23], theoretical optimization [24], or fine separation [25,26,27,28]. To improve workability, advanced mixing [29,30] and warm-mix technologies [31,32,33,34] have been developed to mitigate the conflict between achieving good workability and preventing the secondary aging of RAP at high temperatures [35]. Further attempts include reinforcing mixtures with high-performance additives like epoxy resin [36,37] and steel slag [5,38], or employing refined processing [39,40,41]. However, these solutions often target specific problems in isolation. They may incur high costs, complicate the process, or fail to fully restore the aged skeleton, leading to premature failure. Consequently, a unified framework to simultaneously address the coupled problems of gradation variability, load-bearing capacity, and workability is still lacking.

Specifically, this study developed a novel porous core–shell composite aggregate from RAP fines via controlled cement hydration. This surface modification achieves synergistic effects: (i) stabilizing ‘pseudo-particles’ into ‘true particles’ through mineralization to regulate gradation; (ii) reinforcing the weakened skeleton to enhance load-bearing capacity; and (iii) improving thermomechanical behavior. Furthermore, the study investigated the aggregate’s application in a full-component AC-13 mixture to elucidate the structure–property relationship, demonstrating a viable pathway for high-value RAP utilization in high-grade pavements.

## 2. Materials and Methods

### 2.1. Materials

#### 2.1.1. Reclaimed Asphalt Pavement (RAP)

The RAP used in this study was sourced from the reconstruction and expansion project of the Wu-Huang Expressway in Hubei Province, China. The original pavement, constructed in 2003, was milled for reconstruction in 2024 after 21 years of service without major overhauls. The pavement structure consisted of an 8 cm thick AC-20 binder course and a 4 cm thick Sup-12.5 wearing course. The RAP was not differentiated by its original layer, and its maximum particle size was no larger than 15 mm. The RAP was initially sieved into two fractions, 0–5 mm and 5–15 mm, at the stockpile site, and parameters such as asphalt content and mineral filler content were tested separately. The binder, mineral filler, and aggregates were recovered from the RAP using a centrifugal extraction method. The main technical properties of the RAP, its recovered binder, and reclaimed aggregate are presented in Table 1, Table 2 and Table 3.

#### 2.1.2. Cement

Cement purchased from Yangchun Cement Co., Ltd. in Weifang, Shandong Province, China. Three types of cement with different strength grades were used to treat the RAP. Since ordinary Portland cement 32.5 (PO 32.5) was discontinued in the latest Chinese standard ‘Common Portland cement’ (GB 175-2023) [56], Portland slag cement 32.5 (PS.A 32.5) was used as a substitute. The other two cements were ordinary Portland cement, designated as PO 42.5 and PO 52.5. The technical indicators of cement are shown in Table 4.

### 2.2. Experimental Methods

#### 2.2.1. Preparation of Recycled Aggregates

**Figure 1:**
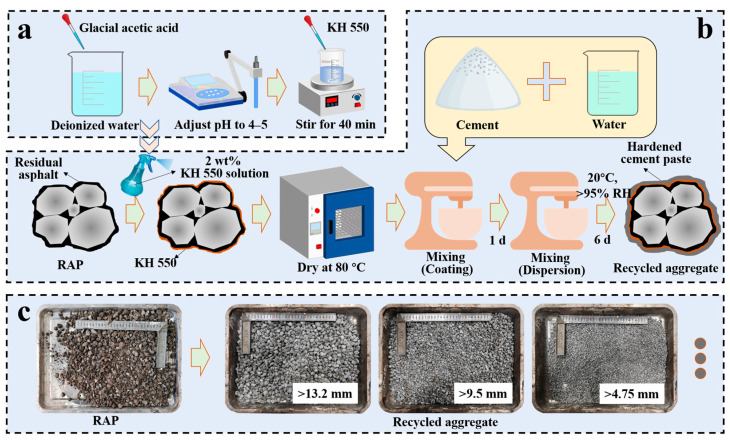
Preparation process of the recycled aggregates: (**a**) preparation of silane coupling agent solution; (**b**) preparation of recycled aggregates; (**c**) macroscopic morphology of RAP and recycled aggregates.

As illustrated in Figure 1, RAP was pre-treated with silane coupling agent (SCA) KH550 to enhance interfacial adhesion. The 2 wt% KH550 solution was prepared by hydrolyzing the agent in deionized water (adjusted to pH 4–5 with glacial acetic acid) for 40 min (Figure 1a). The solution was then sprayed onto RAP at a mass ratio of 0.02 and dried at 80 °C (Figure 1b), followed by mixing with cement and water. To prevent agglomeration, the mixture was remixed after 1 day of curing, followed by final curing (20 °C, relative humidity (RH) > 95%) for 6 days. Finally, the aggregates were sieved for subsequent testing (Figure 1c).

While conducted on a laboratory scale, this workflow is designed to be compatible with existing paving infrastructure. Key steps, such as spray coating and twin-shaft mixing, simulate industrial processes similar to those used in stabilized soil mixing plants, ensuring theoretical feasibility for mass production.

#### 2.2.2. Face-Centered Central Composite Design (FCCD)

FCCD, a specialized response surface methodology (RSM), was employed to model the nonlinear relationships between synthesis parameters and material properties. Unlike standard CCD, FCCD places axial points on the face centers, allowing factors to be evaluated at only three levels, which efficiently reduces experimental runs while maintaining accuracy for second-order fitting. The design involved 20 runs (8 corner, 6 axial, and 6 center points) as listed in Table 5. Design-Expert 11 software was used to perform the FCCD experimental design and data analysis.

**Figure 2:**
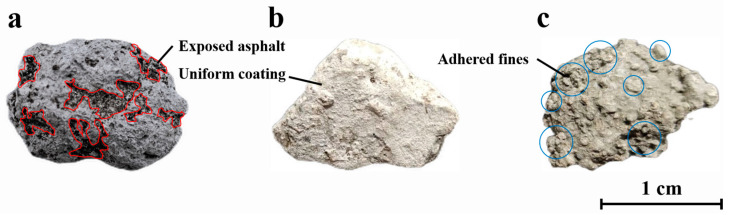
Macroscopic morphology of recycled aggregates with different degrees of cement paste coating: (**a**) undercoated; (**b**) uniformly coated; (**c**) overcoated.

The independent variables were cement strength grade, water–cement ratio, and paste–RAP ratio. Although cement grade is categorical, it was treated as a continuous variable to visualize the strength contribution gradient and construct the prediction model. The water–cement ratio range (0.3–0.5) was based on the literature [57]. The paste–RAP ratio range was determined via preliminary experiments to optimize coating quality. As illustrated in Figure 2, a low ratio leaves exposed asphalt that causes void defects upon heating (Figure 2a), whereas an excessive ratio leads to agglomeration of fines (Figure 2c). The selected range ensures a uniform, robust cementitious shell (Figure 2b).

The selected response variables were crushing value (*CV*), apparent specific gravity (*ASG*), and water absorption (*WA*). These three indicators are among the most critical quantifiable properties for aggregates in asphalt mixtures and are closely correlated with pavement performance. Specifically, the apparent specific gravity and water absorption were determined using three replicates per run, with the average value reported. The crushing value was measured using two replicates; if the deviation exceeded ±10% of the mean, a third test was conducted to ensure accuracy.

#### 2.2.3. Pavement Performance Study

An AC-13 gradation with a target air void content of 4% was used for the pavement performance validation to explore the application potential of the recycled aggregates in high-grade pavements. The aggregate fraction consisted entirely of either RAP or recycled aggregates. Three content levels for the recycled aggregates were investigated: 40%, 70%, and 100%. The recycled aggregates were preferentially used in the coarse aggregate fraction. A new SBS-modified asphalt was used as the binder. When calculating the required amount of SBS-modified asphalt, the mass of the aged asphalt contained in the RAP was deducted. Based on preliminary experiments, the asphalt–aggregate ratio was set at 5.0%, which is the optimal ratio for a conventional AC-13 mixture using SBS-modified asphalt.

The Marshall stability and immersion stability tests were conducted in accordance with ASTM D6927 to evaluate the high-temperature load-bearing capacity and moisture sensitivity of the recycled aggregate asphalt mixtures. Specimens were prepared using Marshall compaction to a height of 63.5 ± 1.3 mm, with compaction blows of 75 and 50 times per side, respectively. Four replicate specimens were tested for each group in the Marshall stability test.

The rutting test was performed according to EN 12697-22 [58] to evaluate the high-temperature anti-rutting performance of the mixtures. Slab specimens with dimensions of 300 mm × 300 mm × 50 mm were fabricated using the wheel-rolling compaction method. Three replicate tests were conducted for the rutting resistance evaluation.

The low-temperature flexural beam test was conducted according to method EN 12697-46 [59] to assess the low-temperature anti-cracking performance of the mixtures. Beam specimens measuring 250 mm × 300 mm × 35 mm were obtained by cutting them from the larger slabs used in the rutting test. Three replicate tests were performed for the low-temperature flexural analysis.

#### 2.2.4. Morphology and Elemental Analysis

A Zeiss Sigma 500 field emission scanning electron microscope (SEM) equipped with an energy-dispersive X-ray spectrometer (EDS) (Carl Zeiss in Oberkochen, Germany) was used to analyze the microscopic morphology and surface element distribution of samples such as RAP and recycled aggregates. Both instruments were operated in low-vacuum mode at appropriate voltages to obtain clear images. The EDS testing employed mapping mode, targeting elements C, O, Al, Si, and Ca, which are closely related to the raw materials or products used in this study, such as basalt, asphalt, cement hydration products, and silane coupling agents.

## 3. Results and Analysis

### 3.1. Optimization of the Preparation Process for Recycled Aggregates

#### 3.1.1. Multi-Objective Response Optimization

Table 6 lists the experimental design and results of the FCCD. As can be seen, the crushing values of the recycled aggregates were generally lower compared to those of the reclaimed aggregates from RAP (22.3%). The higher crushing value of the reclaimed aggregates can be attributed to the effects of repeated traffic loading and the erosion from organic solvents during the extraction process. However, the encapsulating hardened cement paste effectively reconstructed the load-bearing framework of the aggregates, which significantly enhanced their compressive strength. The apparent specific gravity of the recycled aggregates was slightly higher than that of the original RAP (2.33–2.48) but remained below the density levels of reclaimed aggregates (2.67–2.69) or typical virgin aggregates (≥2.70). The water absorption of most recycled aggregates was maintained within 3%. Water absorption is influenced by numerous factors, such as the degree of hydration, the surface roughness of the aggregates, and the density and coverage of the hardened cement paste. Therefore, water absorption serves as a crucial indicator for characterizing the quality of recycled aggregates.

Second-order polynomial models were developed based on the FCCD results (Table 6). To refine the models, non-significant terms (*p* > 0.1) were sequentially removed while adhering to the hierarchical principle. The optimized analysis of variance (ANOVA) results (Table 7, Table 8 and Table 9) exhibit non-significant “Lack of Fit” and high F-values (>10), confirming the models’ validity against background noise. The coefficients of determination (R^2^) for *ASG* and WA exceeded 0.9, indicating high fitting accuracy. The relatively lower R^2^ for *CV* (0.8023) is attributed to the inherent variability of the aggregate crushing test, but remains statistically acceptable. Equations (1)–(3) present the final regression models.

Multi-objective optimization was performed using a desirability function approach based on Equations (1)–(3). The optimization criteria were defined to meet highway surface course specifications: (i) minimize *CV* (weight 4); (ii) maximize *ASG* with a lower limit of 2.60 (weight 5); and (iii) minimize *WA* with an upper limit of 3.0% (weight 3). The assigned weights reflect the stringency of technical standards and their respective impacts on pavement performance.

Table 10 presents the optimal parameters and predicted responses. Given that the theoretical optimal cement grade (49.3 MPa) approximates 52.5 MPa, P.O 52.5 cement was selected for verification. The experimentally prepared aggregates exhibited a *CV* of 12.53%, *ASG* of 2.62, and *WA* of 1.99%, satisfying technical requirements. Notably, the 43.9% reduction in *CV* relative to raw RAP confirms the efficacy of the framework strengthening.

(1) $$CV=81.9058-0.1655A-493.9000C+1010.4000C^{2}$$

(2) $$ASG=0.0169+0.0493A+3.9605B+6.8298C-0.0215AB-4.1000BC-0.0005A^{2}-2.6909B^{2}-10.9636C^{2}$$

(3) $$WA=-10.5930+0.7681A-15.5100B-0.9575C-0.3850AC+60.0000BC-0.0080A^{2}$$

#### 3.1.2. Analysis of Influencing Factors on Recycled Aggregate Preparation

**Figure 3:**
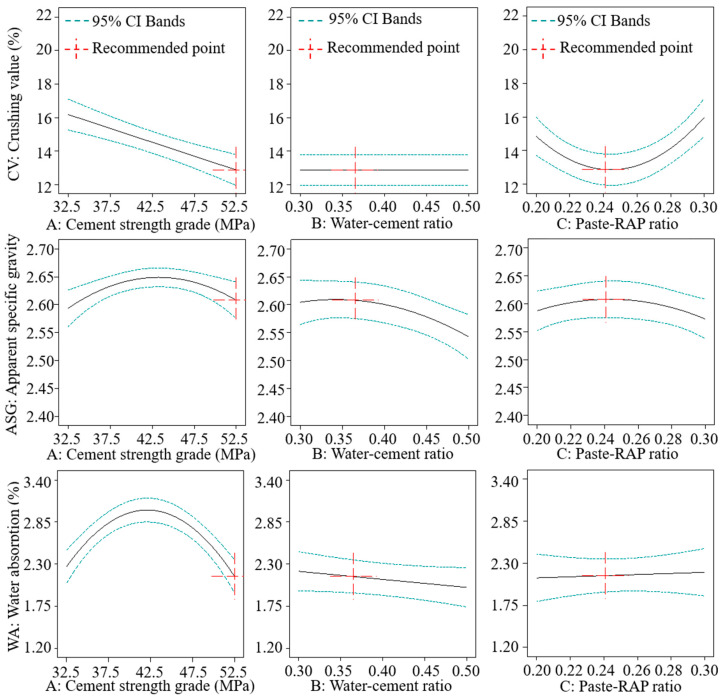
Single-factor effects.

**Figure 4:**
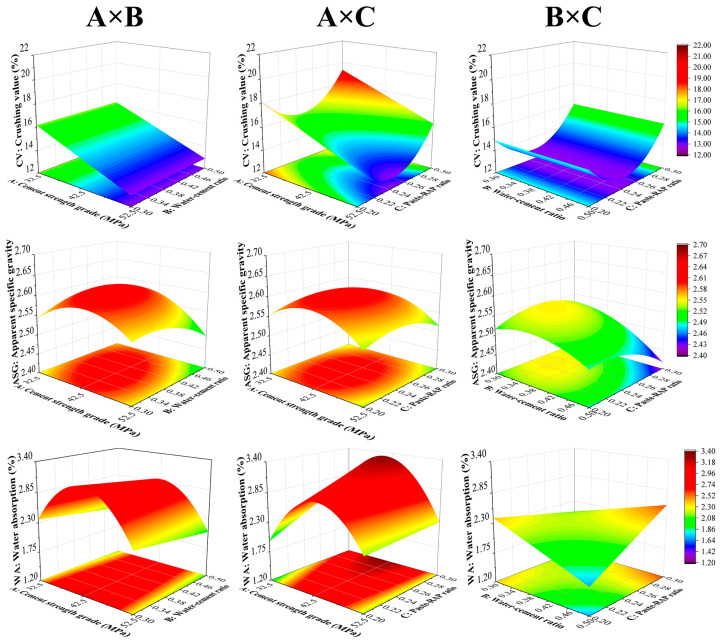
Two-factor interaction effects.

To more clearly understand the effects of the preparation parameters on the properties of the recycled aggregates, an analysis of single-factor effects (Figure 3) and interaction effects (Figure 4) was conducted.

As shown in Figure 3, the *CV* decreased linearly with an increase in cement strength grade, whereas *ASG* and *WA* exhibited a trend of first increasing and then decreasing. The cement strength grade significantly affected the framework strength of the recycled aggregates; thus, a higher-grade cement is undoubtedly more beneficial for reinforcing the RAP. In contrast, *ASG* and *WA* showed a second-order nonlinear response to the cement strength grade. This is primarily because the main effects of cement strength grade on *ASG* and *WA* were not significant (see Table 8 and Table 9). However, different grades of cement often have different optimal water–cement ratios, and the interaction between these two factors likely caused this nonlinear trend.

With an increase in the water–cement ratio, the change in *CV* was not significant. A possible reason is that the *CV* is predominantly governed by the properties of the core RAP and the integrity of the recycled aggregate, making it less sensitive to changes in the water–cement ratio, which mainly affects the strength and density of the hardened cement paste. The *ASG* showed a nonlinear decreasing trend. This is because a higher water–cement ratio leads to more capillary pores in the hardened cement paste, resulting in a more porous and expanded outer shell, which in turn lowers the overall apparent density. The *WA* exhibited a linear decreasing trend, indicating that the cement hydration reaction was more complete at a higher water–cement ratio. If the water–cement ratio is too low, residual unhydrated cement may undergo secondary hydration after hardening, leading to an overestimation of the measured *WA*. Another possible explanation is that a higher water–cement ratio allows for a more uniform coating of the paste, transforming interconnected capillary pores into closed ones and reducing the overall open porosity.

As the paste–RAP ratio increased, the *CV* of the recycled aggregates showed a nonlinear trend of first decreasing and then increasing. The reason is that a low paste–RAP ratio fails to fully coat the RAP surface, preventing the hardened cement paste from forming a complete framework. Additionally, the exposed asphalt on the RAP surface can cause the hardened cement paste to detach under stress, leading to a higher crushing value. However, when the paste–RAP ratio is excessively high, the free cement paste tends to agglomerate fine particles or attach them to the surface of larger particles due to capillary action. This adhesion does not form a complete interlocking framework and is easily broken under external force, causing a significant increase in *CV*. The *ASG* first increased and then decreased, which suggests that the cement paste can effectively fill the surface pores of the RAP and increase the apparent density with its own higher density. However, an excessively high paste–RAP ratio results in porous hardened cement paste from the free paste, leading to a decrease in apparent specific gravity. The *WA* showed a slow, linear increasing trend, but this effect was very limited.

Figure 4 displays the response surface plots for the two-factor interaction effects on the properties of the recycled aggregates. A significant change in the slope of the surface plot as the level of another factor changes indicates a more pronounced interaction between the two corresponding factors. This is visualized in the surface plots as rotated contour lines, twisted surfaces, or asymmetrical surfaces.

For the *CV*, the two-factor interaction response surfaces all exhibited strong symmetry, and the ANOVA showed that the A × B, A × C, and B × C interactions were not significant. Therefore, the response of *CV* to each factor is statistically additive. The curvature observed on each surface primarily stems from the quadratic term of a single factor rather than interaction coupling, and no stable synergistic or antagonistic patterns were observed.

For the *ASG*, the ANOVA indicated that the A × B and B × C interactions were significant, while the A × C interaction was not. Correspondingly, the A × B and B × C surfaces showed features of rotated contour lines or slopes that changed with the other factor, suggesting that the sensitivity of *ASG* to water–cement ratio and paste–RAP ratio is modulated by cement strength grade and water–cement ratio, respectively. It should be emphasized that when both the water–cement ratio and paste–RAP ratio are high, there is an excess of paste and free water. During hardening, this excess free water bleeds and evaporates, easily leaving behind closed pores or entrapped air within the thick paste layer. This increases the apparent volume with limited mass gain, thus causing a significant decrease in *ASG*.

For the *WA*, the A × C and B × C interactions were significant, while the A × B interaction was not. The corresponding surfaces exhibited increased slopes and reduced symmetry. This suggests that the effect of the paste–RAP ratio on *WA* is co-modulated by both the cement strength grade and the water–cement ratio. Specifically, at a lower cement strength grade or a higher water–cement ratio, increasing the paste–RAP ratio is more likely to amplify the *WA*. Appropriately increasing the cement strength grade can partially mitigate this adverse effect. Therefore, controlling *WA* should focus on the synergistic regulation between the paste–RAP ratio and the other two factors, rather than simply pairing the cement strength grade with its “optimal” water–cement ratio.

### 3.2. Analysis of the Particle Size Regulation Mechanism

A key advantage of preparing recycled aggregates from fine RAP material is the significant increase in particle size after treatment, which effectively addresses the issue of excess fine materials. Therefore, the key to preparing recycled aggregates lies in understanding the factors and mechanisms that influence their particle size to achieve controllable regulation. Further quantitative analysis is beneficial for the engineering implementation of particle size-controlled recycled aggregates. Accordingly, three quantitative indicators were defined to evaluate the degree of gradation optimization of the recycled aggregates: Median particle size increment (ΔD_50_): The difference in sieve sizes corresponding to 50% passing, calculated through linear interpolation, reflecting the change in the overall framework particle size. Fine material reduction (ΔP_4.75_): The difference in the passing rate through the 4.75 mm sieve before and after treatment, directly reflecting how much fine material was converted into coarse aggregate during the recycling process. Gradation adjustment index (GAI): The integral of the “difference in passing rates” with respect to the “logarithmic sieve size.” It represents a weighted sum of the “increment of particles retained on the sieve” across all sieve sizes, which can be calculated using Equation (4) [29]:

(4) $$GAI=\int_{D_{min}}^{D_{max}}\left|P_{A}\left(D\right)-P_{B}\left(D\right)\right|d\left(\log_{10}D\right)$$

where D_max_ is the maximum sieve size; D_min_ is the minimum sieve size; and P_A_(D) and P_B_(D) are the passing rates (%) for gradations A and B at sieve size D, respectively.

In Table 6, the cement strength grade for experimental runs No. 9, No. 15, and No. 10 increases sequentially, while the other two variables are controlled at their medium levels. Therefore, by conducting sieve analysis on these three groups of recycled aggregates, the effect of cement strength grade on the particle size of the recycled aggregates can be determined. The effects of the other two variables can be obtained in a similar manner.

**Figure 5:**
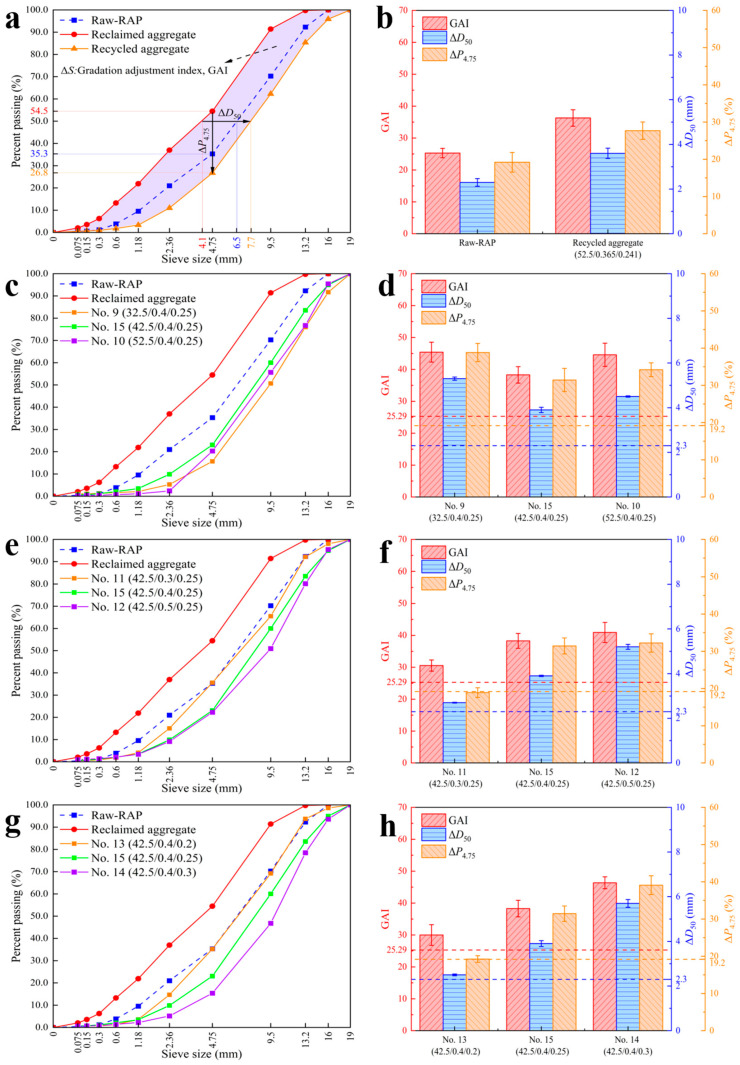
Effects of preparation conditions on the gradation of recycled aggregates: (**a**) recommended scheme; (**b**) quantitative analysis of the recommended scheme; (**c**) cement strength grade; (**d**) uantitative analysis of cement strength grade; (**e**) water–cement ratio; (**f**) quantitative analysis of water–cement ratio; (**g**) paste–RAP ratio; (**h**) quantitative analysis of paste–RAP ratio.

Figure 5a,b show the effects of different preparation factors on the particle size and gradation composition of the recycled aggregates. Compared to the original RAP (raw-RAP), the gradation curve of the reclaimed aggregates shifted significantly to the left and upward, indicating that the proportion of fine aggregate in the RAP is much higher than the sieving results suggest. For the recycled aggregates, the gradation curves showed a trend of shifting to the right and downward, which demonstrates that the particle size of the recycled aggregates was effectively increased. The “apparent particle size” and “apparent gradation” of the RAP were transformed into the “true particle size” and “true gradation” of the recycled aggregates. The ΔD_50_ and ΔP_4.75_ of the raw-RAP were 2.3 mm and 19.2%, respectively, meaning that the actual median particle size of the RAP was 2.3 mm larger than that of the reclaimed aggregate, and the sieved content of fine materials below 4.75 mm was 19.2% lower than the actual value. For the recycled aggregates, the median particle size was 3.6 mm larger than the actual value of the reclaimed aggregate, and the fine aggregate content was reduced by 27.69%. The GAI of the recycled aggregates reached 1.43 times that of the RAP, indicating that the coating treatment had a significant effect on improving the gradation variability of the RAP.

Figure 5c,d show that as the cement strength grade increased, the overall shapes of the gradation curves for the recycled aggregates were similar. The differences were mainly observed in the 4.75–13.2 mm range but were very small in magnitude. The corresponding GAI, ΔD_50_, and ΔP_4.75_ indicators showed no significant differences and did not exhibit a monotonic trend with cement strength grade. A potential reason for the low sensitivity of the gradation to cement strength grade is the difference in composition between P.S.A 32.5 and P.O 42.5/52.5 cements. Differences in chemical–mineralogical composition and early hydration behavior may introduce systematic deviations. The minor differences in the gradation curves are more likely due to variations in cement type and operational errors rather than the cement strength grade itself. Therefore, within the scope of this study, cement strength grade is considered a secondary factor affecting gradation.

Figure 5e,f show that as the water–cement ratio increased, the particle size of the recycled aggregates slightly increased, with the curves shifting overall to the right and downward. The fine material content decreased by 18.91%, 31.44%, and 32.24%, respectively, compared to the reclaimed aggregate. The median particle sizes increased by 2.7 mm, 3.9 mm, and 5.2 mm, and the GAI values were 30.54, 38.28, and 40.91, all showing a monotonic upward trend. However, at a water–cement ratio of 0.3, the passing rates for particle sizes above 4.75 mm were almost indistinguishable from those of the raw-RAP, while the passing rates for 2.36 mm and below were relatively lower. This could be because cement hydration was incomplete under this condition, resulting in a smaller volume and higher yield stress of the paste, which existed more as a thin film coating. The thickness of this cement film had almost no effect on the passing rates for sieve sizes above 4.75 mm, but significantly affected the passing rates for smaller sieves. As the water–cement ratio increased to 0.4, this phenomenon was mitigated. However, as it further increased to 0.5, the passing rates for sieves of 4.75 mm and below no longer increased, suggesting that an optimal water–cement ratio exists that allows for a uniform increase across all particle size fractions.

Figure 5g,h show that the higher the paste–RAP ratio, the more significant the increase in the particle size of recycled aggregates. However, when the paste–RAP ratio was at a low level, its effect on increasing the passing rates for sieves of 4.75 mm and above was also very limited, with a ΔP_4.75_ of 19.25%, almost identical to that of the raw-RAP. This is similar to the case of the water–cement ratio: if the amount of cement paste is too small, it cannot fully coat the RAP or can only form a thin layer, which significantly affects the passing rates of small-sized sieves but hardly changes those of large-sized sieves. As the paste–RAP ratio increased to 0.3, the GAI, ΔD_50_, and ΔP_4.75_ of the recycled aggregates reached 46.37, 5.7 mm, and 39.1%, respectively. This was the most significant change among all samples, indicating that the paste–RAP ratio plays a decisive role in regulating the particle size and gradation of recycled aggregates.

### 3.3. Analysis of Pavement Performance

**Figure 6:**
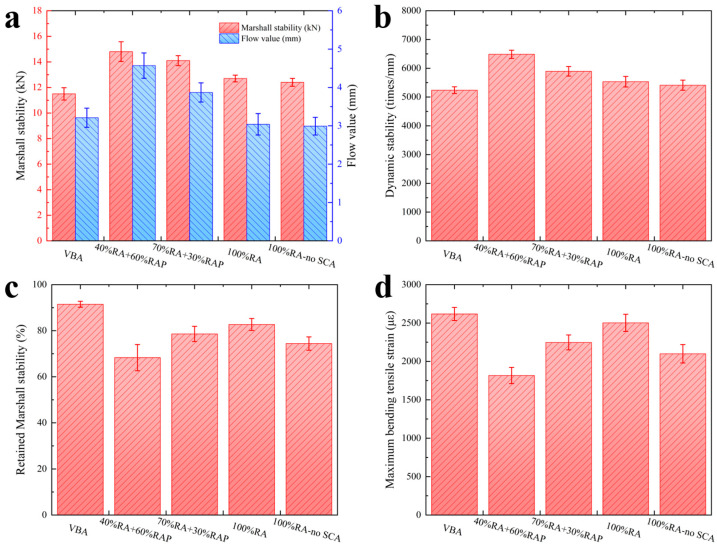
Pavement performance validation: (**a**) Marshall stability and flow value; (**b**) dynamic stability; (**c**) retained Marshall stability; (**d**) maximum bending tensile strain.

As shown in Figure 6a, Marshall stability gradually decreased as the proportion of recycled aggregates (RA) increased, yet all mixtures significantly outperformed the virgin basalt aggregate (VBA) control. The 100% RA mixture achieved 110.4% of the VBA stability with lower flow, indicating superior deformation resistance. Conversely, mixtures with high RAP content (e.g., 40% RA + 60% RAP) exhibited higher stability due to aged asphalt stiffness but suffered from excessive flow, likely caused by weak adhesion at the “aged binder-new binder” interface. Since the flow value of the 40% RA group exceeded specification limits (JTG F40-2004), a minimum RA content of 70% is recommended. The KH550 coupling agent showed a negligible impact on Marshall metrics.

The trend of dynamic stability shown in Figure 6b was similar to that of Marshall stability. When 100% recycled aggregates were used, the dynamic stability reached 105.7% of that of the virgin basalt aggregate mixture. At lower relative contents of RA, the dynamic stability of the mixture was higher, suggesting that, in terms of high-temperature performance, the high modulus of the aged asphalt in the high-content RAP was the dominant factor, and its contribution surpassed the framework-strengthening effect of the recycled aggregates. The dynamic stability of the 100% recycled aggregate mixture without the coupling agent was almost unaffected, remaining within the deviation range of the coupled group and slightly higher than the basalt aggregate mixture.

As shown in Figure 6c, the retained Marshall stability for all recycled mixtures was lower than the control. The 40% RA mixture failed to meet specifications at only 68.3%. Although stability improved with higher RA content, it remained below 85%, limiting its application in humid regions. This deficiency is attributed to two factors: (i) the complex multiphase interfaces within the composite and (ii) the inherent hydrophilicity of the cementitious shell. Hydration products, rich in polar hydroxyl groups and high surface energy, have a stronger affinity for water than non-polar asphalt. Water preferentially wets the shell surface, displacing the asphalt film and causing stripping. To mitigate this, future strategies should focus on hydrophobic modification of the shell or the use of anti-stripping agents (e.g., hydrated lime).

As shown in Figure 6d, the maximum bending tensile strain increased with RA content. The core–shell structure progressively replaces the weak “aged binder–new binder” interface with a stronger “cement shell–new binder” interface, reinforced by the coupling agent. This shell physically isolates the brittle RAP core from the matrix, facilitating stress transfer and mitigating aged binder embrittlement. However, the strain of the 100% RA group remained lower than the control. This is likely due to the modulus mismatch between the rigid cement shell and the viscoelastic asphalt, creating stress concentration zones that serve as crack initiation sites. Further optimization of the mix design (e.g., asphalt content) is required to address this limitation.

### 3.4. Surface Characteristics and Elemental Analysis

**Figure 7:**
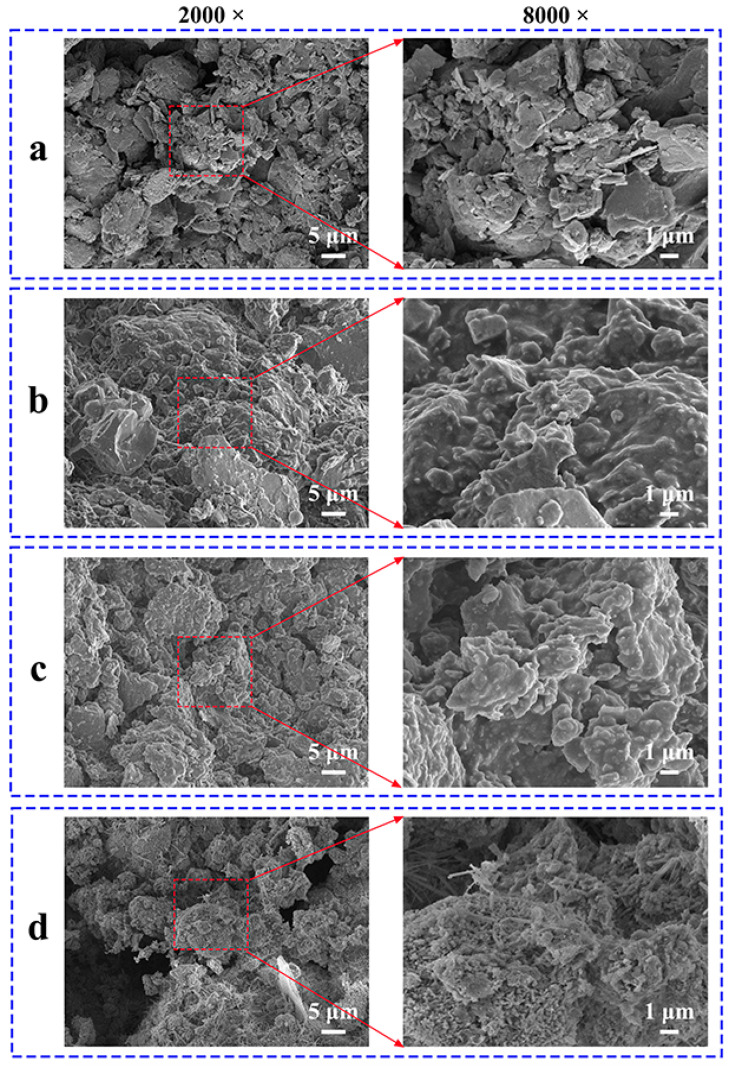
Surface morphology of aggregates: (**a**) basalt aggregate; (**b**) RAP; (**c**) RAP treated with coupling agent; (**d**) recycled aggregate.

The surface morphology of aggregates is closely related to their adhesion properties. Figure 7 presents the scanning electron microscopy (SEM) images of different aggregates. The surface of the basalt aggregate was rough, and some flaky particles provided the basalt with a larger specific surface area, which is beneficial for its bonding with asphalt. The surface morphology of the RAP changed significantly; due to the asphalt coating, the angularity and roughness of the aggregate surface were markedly reduced. Treatment with the KH550 coupling agent did not cause a significant change in morphology. The surface morphology of the recycled aggregates changed dramatically. First, a large number of loose and porous agglomerates were formed on the surface, which are calcium silicate hydrate (C-S-H) gels, the main component and primary strength contributor in hardened cement paste. In addition, at 8000× magnification, many needle-like crystals could be clearly seen, which are ettringite (AFt), one of the main products of early cement hydration. These features significantly increased the surface roughness of the RAP.

**Figure 8:**
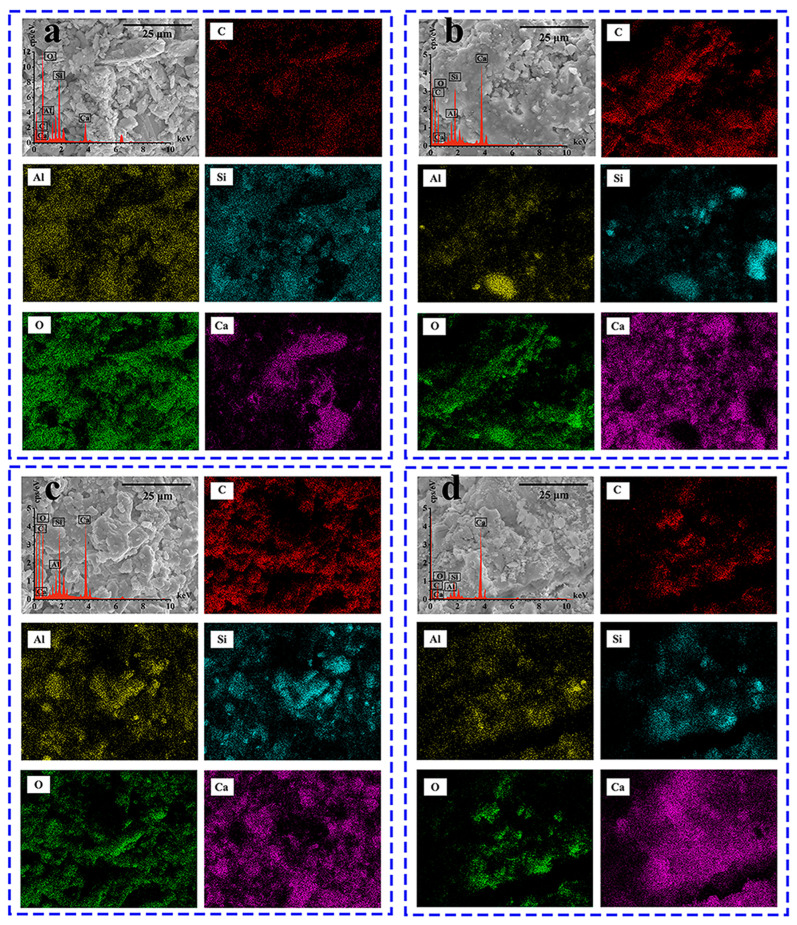
EDS test results of aggregates: (**a**) basalt aggregate; (**b**) RAP; (**c**) RAP treated with coupling agent; (**d**) recycled aggregate.

**Figure 9:**
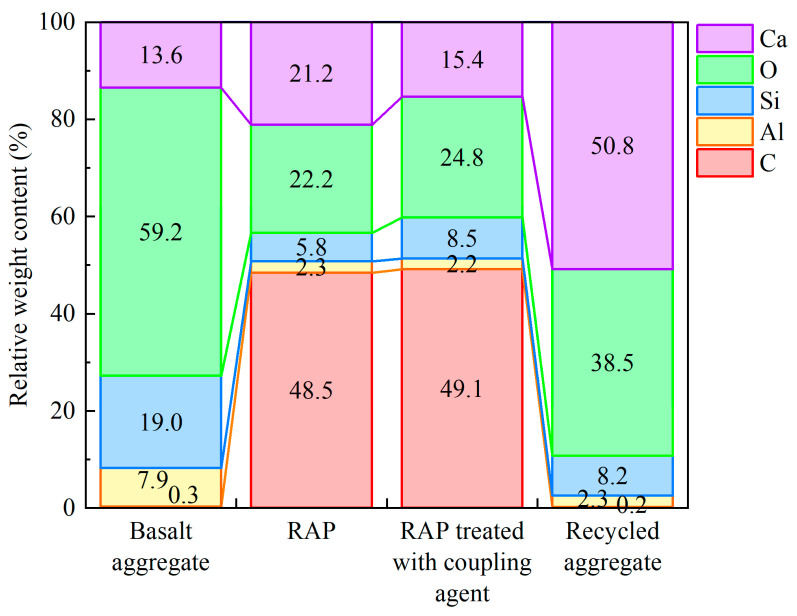
Stacked bar chart of relative elemental weight content from EDS.

Figure 8 shows the EDS test results for the four types of aggregates. To provide a quantitative assessment of the surface modification mechanism, the relative weight contents of key elements were analyzed, and the results are shown in Figure 9. The main components of basalt are silica and alumina, characterized by a dominant presence of O (59.2%) and Si (19.0%), with negligible C content (0.3%). For the RAP sample, the detection of C surged to 48.5%, becoming the primary surface element. This dramatic shift in the C/Si ratio (from ~0.02 in basalt to ~8.36 in RAP) quantitatively confirms the substantial coverage of the organic asphalt binder on the mineral aggregate. The significant presence of Ca (21.2%) was mainly derived from the limestone mineral filler in the asphalt mixture. The chemical formula of the KH550 coupling agent is C_9_H_23_NO_3_Si. As shown in Figure 8c, while the C content remained at a high level (49.2%), the Si content exhibited a notable increase from 5.8% (in RAP) to 8.5%. This relative increase of approximately 46% in silicon concentration provides robust evidence that the silane coupling agent containing Si functional groups was successfully grafted onto the RAP surface. Regarding the recycled aggregates, the surface chemical composition underwent a fundamental reversal. The most abundant element was Ca (reaching 50.8%), while the C content plummeted to 0.2%. The high Ca/Si ratio (~6.2) combined with the dominant O content (38.5%) indicates that the tested area is composed of calcium-rich hydration products, such as Ca(OH)_2_ crystals and C-S-H gel. The cement coating can effectively improve the surface morphology and chemical properties of the RAP recycled aggregates, providing a micro-level basis for their efficient reuse in recycled asphalt mixtures.

## 4. Discussion

### 4.1. Sustainability Analysis

A qualitative assessment suggests the proposed method yields positive net environmental benefits. Although Portland cement (cement-to-RAP mass ratio ≈ 0.176) and a silane coupling agent (≈0.04%) are introduced, these controlled dosages enable the 100% recycling of RAP fines. This effectively offsets the substantial energy consumption and ecological impact associated with mining, crushing, and transporting virgin aggregates. Regarding carbon emissions, while cement increases the initial embodied carbon, this is compensated for from a lifecycle perspective by eliminating RAP stockpiling and extending pavement service life, thereby reducing maintenance frequency and its associated carbon footprint.

### 4.2. Limitations

First, the method was validated using a single RAP source and mixture type. Given the significant variability in RAP due to milling origins and aging history, the robustness of the core–shell synthesis warrants validation across diverse material types. Second, long-term durability data—specifically fatigue behavior under complex coupled environmental-load conditions and field performance—are currently lacking. Third, for ease of comparison, the road performance tests of this study were conducted based on the same asphalt dosage, which may have greatly limited the performance of recycled asphalt mixtures. Finally, the regression model simplifies the cement strength grade as a continuous variable for trend visualization. Due to inherent RAP variability, this model reflects average response behaviors rather than precise predictions, serving primarily as an engineering reference.

### 4.3. Future Perspectives

Future research should expand the experimental scope to include RAP sources with varying lithologies and aging degrees to verify the method’s universality. To address limitations such as moisture stability, optimizing mix designs or applying secondary modification treatments is recommended. Additionally, while this study used standard cement to validate the preparation framework, future work could employ low-carbon binders (e.g., geopolymers or resins) to further enhance environmental benefits.

## 5. Conclusions

This study systematically investigated the performance enhancement and particle size regulation effects of cement treatment of reclaimed asphalt pavement (RAP). A face-centered central composite design (FCCD) was employed to optimize the preparation process. The simultaneous strengthening effects and particle size regulation mechanisms of this process on RAP were analyzed. Furthermore, full-component recycled asphalt mixtures were prepared to validate the application potential of the recycled aggregates in high-grade pavements. Based on pavement performance tests and microstructural characterization, the following main conclusions were drawn:

(1) The cement strength grade plays a decisive role in the strengthening effect on the recycled aggregates, exhibiting a significant linear negative correlation with the crushing value. The paste–RAP ratio showed significant second-order nonlinear effects on both the crushing value and apparent specific gravity, indicating the existence of an optimal coating amount to achieve the best framework structure and densification. Although the direct effect of the water–cement ratio was not prominent, it played a key regulatory role in the final properties of the aggregates through its significant interactions with the cement strength grade and paste–RAP ratio.

(2) High-performance cement-treated recycled aggregates were successfully prepared by optimizing the process using FCCD. Compared to the original reclaimed aggregate, the crushing value of the recycled aggregates was reduced by 43.9%. Other key indicators, such as apparent specific gravity and water absorption, met the technical requirements for coarse aggregates used in highway surface courses. This demonstrates that the proposed technology can effectively restore and enhance the engineering properties of RAP.

(3) Compared to the original reclaimed aggregate, the median particle size (D_50_) of the recycled aggregates increased by 3.6 mm, and the content of fine materials below 4.75 mm decreased by 27.69%. This process effectively transformed fine aggregates into coarse aggregates, mitigating the issues of gradation variability and excess fine materials common in RAP recycling applications. The primary factor influencing the particle size of the recycled aggregates was the paste–RAP ratio, followed by the water–cement ratio, while the cement strength grade did not have a significant effect.

(4) In full-component recycled asphalt mixtures, a high relative content of RAP, while enhancing high-temperature stability, had adverse effects on low-temperature cracking resistance and moisture stability. Increasing the proportion of cement-treated recycled aggregates in the total aggregate blend can effectively compensate for these performance deficiencies. The application of a silane coupling agent significantly strengthened the weak interface between the aged asphalt and the cement-treated shell, thereby further enhancing the ability of the recycled aggregates to resist moisture damage. This study achieved the application of full-component RAP in AC-13 mixtures, provided that at least 70% of the aggregates consist of core–shell cement recycled aggregates.

(5) SEM and EDS analyses revealed that cement hydration products, such as calcium silicate hydrate (C-S-H) gel and ettringite (AFt), formed a mineralized shell on the surface of the RAP particles. This shell not only significantly increased the surface roughness of the RAP but also modified its surface chemical properties, providing a solid physical and chemical foundation for enhancing the interfacial adhesion with new asphalt.

## Figures and Tables

**Table 1:** Main technical properties of RAP.

Test Item		Measured Value	Technical Requirements (JTG/T 5521-2019 [42])	Test Method
Moisture content (%)		0.81	≤3	ASTM D2216 [43]
Sand equivalent		69	≥60	ASTM D2419 [44]
0–5 mm	Asphalt content (%)	5.92	-	ASTM D2172 [45]
	Mineral filler content (%)	8.14	-	ASTM D5444 [46]
	Apparent specific gravity	2.33	-	ASTM C128 [47]
5–15 mm	Asphalt content (%)	3.51	-	ASTM D2172
	Mineral filler content (%)	1.41	-	ASTM D5444
	Apparent specific gravity	2.48	-	ASTM C127 [48]

**Table 2:** Main technical properties of recovered asphalt from RAP.

Test Item	Measured Value	Technical Requirements (JTG F40-2004 [49], SBS I-D)	Test Method
Penetration (0.1 mm)	34.4	40~60	ASTM D5 [50]
Softening point (°C)	76.5	≥60	ASTM D36 [51]
Ductility at 15 °C (cm)	32.8	-	ASTM D113 [52]
Ductility at 5 °C (cm)	8.4	≥20	ASTM D113

**Table 3:** Main technical properties of reclaimed aggregates from RAP.

Test Item		Measured Value	Technical Requirements (JTG F40-2004, Expressway Surface Layer)	Test Method
Crushing value (%)		22.3	≤26	BS 812-110 [53]
Los Angeles abrasion loss (%)		13.5	≤28	ASTM C131 [54]
Flat and elongated particles (%)		5.7	≤15	ASTM D4791 [55]
0–5 mm	Apparent specific gravity	2.69	≥2.50	ASTM C128
5–15 mm	Apparent specific gravity	2.67	≥2.60	ASTM C127

**Table 4:** Main technical properties of cement.

Test Item	PS.A 32.5	PO 42.5	PO 52.5
SO_3_ (%)	2.23	2.12	1.98
Cl-content (%)	0.035	0.015	0.046
True density (g/cm^3^)	3.148	3.155	3.153
Fineness (>0.08 mm) (%)	2.6	1.8	2.5
Initial setting time (min)	225	186	182
Final setting time (min)	248	234	304
3d flexural strength (MPa)	3.1	3.6	6.2
3d compressive strength (MPa)	15.2	21.3	31.2
28d flexural strength (MPa)	6.2	8.3	9.4
28d compressive strength (MPa)	38.8	49.1	61.4

**Table 5:** Factors and levels of the FCCD.

Factors		Code	Unit	Low Level	Medium Level	High Level
Independent variables	Cement strength grade	*A*	MPa	32.5	42.5	52.5
	Water–cement ratio	*B*	-	0.3	0.4	0.5
	Paste–RAP ratio	*C*	-	0.2	0.25	0.3
Response variables	Crushing value	*CV*	%	-	-	-
	Apparent specific gravity	*ASG*	-	-	-	-
	Water absorption	*WA*	%	-	-	-

**Table 6:** FCCD experimental design and results.

Run	Cement Compressive Strength (MPa)	Water–Cement Ratio	Paste–RAP Ratio	Crushing Value (%)	Apparent Specific Gravity	Water Absorption (%)
1	32.5	0.3	0.2	18.15	2.53	2.34
2	52.5	0.3	0.2	14.21	2.59	2.14
3	32.5	0.5	0.2	17.62	2.61	1.23
4	52.5	0.5	0.2	16.43	2.54	1.75
5	32.5	0.3	0.3	18.92	2.55	2.55
6	52.5	0.3	0.3	15.06	2.58	2.20
7	32.5	0.5	0.3	20.45	2.51	3.26
8	52.5	0.5	0.3	16.97	2.49	2.39
9	32.5	0.4	0.25	16.82	2.58	2.25
10	52.5	0.4	0.25	12.74	2.58	2.16
11	42.5	0.3	0.25	13.68	2.61	3.15
12	42.5	0.5	0.25	13.11	2.60	3.24
13	42.5	0.4	0.2	16.13	2.61	2.84
14	42.5	0.4	0.3	16.79	2.60	3.24
15	42.5	0.4	0.25	16.23	2.65	3.12
16	42.5	0.4	0.25	15.12	2.63	2.74
17	42.5	0.4	0.25	14.28	2.66	3.25
18	42.5	0.4	0.25	13.55	2.65	2.99
19	42.5	0.4	0.25	15.31	2.69	2.69
20	42.5	0.4	0.25	14.63	2.66	3.03

**Table 7:** ANOVA results for the crushing value after model optimization.

Source	Sum of Squares	Degrees of Freedom	Mean Square	F-Value	*p*-Value	
Model	62.49	3	20.83	21.64	<0.0001	significant
A-Cement strength grade	27.39	1	27.39	28.46	<0.0001	significant
C-Paste–RAP ratio	3.19	1	3.19	3.32	0.0873	not significant
C^2^	31.90	1	31.90	33.15	<0.0001	significant
Residual	15.40	16	0.9624			
Lack of fit	11.15	11	1.01	1.19	0.4511	not significant
Pure error	4.25	5	0.8504			
Corrected total	77.88	19				
R^2^	0.8023					

**Table 8:** ANOVA results for the apparent specific gravity after model optimization.

Source	Sum of Squares	Degrees of Freedom	Mean Square	F-Value	*p*-Value	
Model	0.0498	8	0.0062	13.29	0.0001	significant
A-Cement strength grade	9 × 10^−7^	1	9 × 10^−7^	0.0019	0.9658	not significant
B-Water–cement ratio	0.0017	1	0.0017	3.66	0.0820	not significant
C-Paste–RAP ratio	0.0021	1	0.0021	4.55	0.0563	not significant
AB	0.0037	1	0.0037	7.89	0.0170	significant
BC	0.0034	1	0.0034	7.18	0.0214	significant
A^2^	0.0063	1	0.0063	13.48	0.0037	significant
B^2^	0.0020	1	0.0020	4.25	0.0637	not significant
C^2^	0.0021	1	0.0021	4.41	0.0596	not significant
Residual	0.0052	11	0.0005			
Lack of fit	0.0033	6	0.0006	1.49	0.3401	not significant
Pure error	0.0019	5	0.0004			
Corrected total	0.0550	19				
R^2^	0.9062					

**Table 9:** ANOVA results for the water absorption after model optimization.

Source	Sum of Squares	Degrees of Freedom	Mean Square	F-Value	*p*-Value	
Model	5.47	6	0.9120	20.23	<0.0001	significant
A-Cement strength grade	0.0980	1	0.0980	2.17	0.1642	not significant
B-Water–cement ratio	0.0260	1	0.0260	0.5769	0.4611	not significant
C-Paste–RAP ratio	1.12	1	1.12	24.74	0.0003	significant
AC	0.2965	1	0.2965	6.58	0.0235	significant
BC	0.7200	1	0.7200	15.97	0.0015	significant
A^2^	3.22	1	3.22	71.34	<0.0001	significant
Residual	0.5861	13	0.0451			
Lack of fit	0.3499	8	0.0437	0.9258	0.5618	not significant
Pure error	0.2362	5	0.0472			
Corrected total	6.06	19				
R^2^	0.9033					

**Table 10:** Optimized process parameters.

Value	Cement Strength Grade (MPa)	Water–Cement Ratio	Paste–RAP Ratio	Crushing Value (%)	Apparent Specific Gravity	Water Absorption (%)
Predicted	49.3	0.365	0.241	13.40	2.63	2.59
Actual	52.5	0.365	0.241	12.53	2.62	1.99

## Data Availability

The original contributions presented in this study are included in the article. Further inquiries can be directed to the corresponding authors.
